# Imaging diagnosis of cystic intraparenchymal brain neoplasms

**DOI:** 10.1007/s11604-026-02001-w

**Published:** 2026-05-30

**Authors:** Sonoko Oshima, Yasutaka Fushimi, Sachi Okuchi, Satoshi Nakajima, Akihiko Sakata, Takayuki Yamamoto, Yuji Nakamoto, Noriko Salamon

**Affiliations:** 1 Department of Diagnostic Imaging and Nuclear Medicine, Graduate School of Medicine, Kyoto University, Kyoto, Japan; 2 Department of Radiological Sciences, David Geffen School of Medicine, University of California, Los Angeles, Los Angeles, CA USA; 3 Biomedical Imaging Research Center, University of Fukui, Fukui, Japan

**Keywords:** Central nervous system, Intraparenchymal tumor, Cystic brain lesion, Magnetic resonance imaging

## Abstract

Certain brain tumors exhibit cystic components. MRI is particularly effective in characterizing the location, enhancement, and morphology of these cystic regions, enabling radiologists to narrow the differential diagnosis. Cystic components are often associated with circumscribed tumors and may be linked to favorable outcomes. High-grade tumors such as glioblastoma can also occasionally exhibit cystic components. Here, we summarize the imaging characteristics of representative cystic brain intraparenchymal neoplasms, including hemangioblastoma, pilocytic astrocytoma, pleomorphic xanthoastrocytoma, ganglioglioma, desmoplastic infantile ganglioglioma/astrocytoma, central neurocytoma, dysembryoplastic neuroepithelial tumor, ependymoma, glioblastoma, and metastases. This review aims to provide MRI features that facilitate differentiation of these tumors and support appropriate clinical management.

**Supplementary Information:**

The online version contains supplementary material available at https://doi.org/10.1007/s11604-026-02001-w.

## Introduction

Certain brain tumors demonstrate cystic components either within the tumor mass or as surrounding fluid-filled compartments. Accurate radiologic evaluation provides detailed insights into cystic and solid components, and specific imaging patterns can help narrow the differential diagnosis. Cystic components are commonly observed in certain low-grade neoplasms such as pilocytic astrocytoma (PA) and glioneuronal tumors, in association with an expansive, well-circumscribed growth pattern. High-grade tumors such as glioblastoma may also exhibit cystic components.

This review provides a comprehensive overview of both conventional and advanced MR imaging characteristics of intraparenchymal brain neoplasms that typically contain cystic components, as classified according to the 2021 WHO criteria. These include lesions presenting as cysts with mural nodules, such as hemangioblastoma (HB), PA, pleomorphic xanthoastrocytoma (PXA), ganglioglioma, and desmoplastic infantile ganglioglioma/astrocytoma (DIG/DIA). Multiloculated or “soap-bubble-like” tumors, including dysembryoplastic neuroepithelial tumor (DNET) and central neurocytoma, are also discussed. In addition, ependymoma, which often demonstrates a solid mass with cystic components, is reviewed. Finally, tumors characterized by irregular cystic or necrotic changes, such as glioblastoma and brain metastasis, are included (Table 1). Although other glioneuronal tumors, such as papillary glioneuronal tumor, rosette-forming glioneuronal tumor, and myxoid glioneuronal tumor, can also present with cystic components, these relatively rare entities are beyond the scope of this review. By highlighting key imaging features of these lesions, this article aims to assist radiologists in making more accurate interpretations and to support informed clinical decision-making.

**Table 1:** Clinical and Imaging characteristics of cystic brain tumors

Morphologic pattern	Tumor type	Typical age group	Common location	Enhancement pattern	Other imaging features (including DWI/PWI)
Cyst with mural nodule	Hemangioblastoma (HB)	Adults (especially with VHL)	Cerebellum, brainstem, and spinal cord	Intense enhancement of mural nodule; no enhancement on cyst wall	Flow voids on T2WI; high ADC; high rCBV
	Pilocytic Astrocytoma (PA)	Children / Adolescents	Cerebellum, optic pathway (especially with NF1), and hypothalamus	Intense enhancement of mural nodule; cyst wall sometimes enhances	High ADC
	Pleomorphic Xanthoastrocytoma (PXA)	Children / Young adults	Cerebral cortex (temporal > frontal/parietal lobes)	Variable wall/nodule enhancement	Leptomeningeal enhancement; occasional calcification; minimal edema
	Ganglioglioma	Children / Young adults	Temporal lobe > other lobes, brainstem, and cerebellum	Variable (absent to minimal, ring-like, or diffuse)	Occasional calcification; minimal edema; association with focal cortical dysplasia
	Desmoplastic Infantile Ganglioglioma / Astrocytoma (DIG/DIA)	Infants (< 2 years)	Frontal and parietal lobes	Intense enhancement of solid component	Large lesion; Dural-based mural nodule; minimal edema
Multiloculated “bubbly” lesion	Dysembryoplastic Neuroepithelial Tumor (DNET)	Children / Adolescents	Cerebral cortex (temporal > frontal/parietal lobes)	Mild or absent	High ADC; low rCBV; minimal edema; association with focal cortical dysplasia
	Central Neurocytoma	Young adults (20–40 years)	Lateral ventricles (foramen of Monro)	Moderate, heterogeneous	Frequent calcification; scalloping sign; ↑glycine and alanine on MRS
Solid mass with cystic components	Ependymoma	Children to young adults	Regions adjacent to the fourth and lateral ventricles	Heterogeneous	Frequent calcification, hemorrhage, variable edema
	Glioblastoma	Adults	Cerebral hemispheres	Irregular ring-enhancing mass with ill-defined margins	Extensive edema; low ADC and high rCBV in solid portion
	Brain Metastasis	Adults with systemic malignancy	Variable	Peripheral rim enhancement	Multiple lesions; Marked edema; high rCBV at enhancing rim

## Formation of cystic components

The mechanisms underlying cyst formation in brain tumors remain incompletely understood, but several mechanisms have been proposed [1, 2].

One important factor is a vasogenic process. Disruption of the blood-brain barrier allows plasma proteins and other macromolecules to leak into the interstitial space, leading to peritumoral edema. Histopathologic and protein gradient studies have demonstrated that the majority of cyst fluid proteins are derived from plasma rather than endogenous cerebral proteins, supporting the role of blood-brain barrier disruption in cyst fluid composition [1–4]. In this context, this protein-rich environment may lead to elevated amide proton transfer-weighted signal intensity within cystic components, representing a potential pitfall in chemical exchange saturation transfer (CEST)-based tumor assessment [5]. Also, cerebrospinal fluid (CSF) entrapment has been proposed as a potential mechanism involved in cyst development [6].

The capacity of the surrounding tissue to absorb excess fluid is also considered to influence cyst formation [2–4]. Edema fluid is cleared either by drainage into the CSF or by degradation through reactive glial elements. If the balance between fluid accumulation and reabsorption becomes insufficient, microcystic spaces may progressively merge into larger cavities. Once larger cavities form, their low surface-to-volume ratio may hinder reabsorption through the cyst wall. Histological examination of the tumor cysts reveals a gradual liquefaction of edematous, spongy tissue, resulting in the accumulation of cyst fluid, while other regions of the wall display a sharply demarcated cellular lining, which is thought to reflect areas where the cyst has enlarged through expansion and compression of the surrounding parenchyma [2].

In addition to these mechanisms, necrosis may also result in fluid-containing spaces within tumors. As neoplastic tissue outgrows its blood supply, limited vascular density and increased cell-to-vessel distances result in insufficient oxygen and nutrient delivery. This hypoxic and ischemic microenvironment leads to cellular death and reduced glucose metabolism, with elevated lactate levels observed in cyst fluid. As a consequence, both necrotic tissue and edematous parenchyma may undergo gradual liquefaction, forming fluid-filled spaces within the tumor core. Furthermore, microvascular events such as thrombosis or hemorrhage may generate small degenerative foci that evolve into microcysts, which subsequently enlarge and merge to form macroscopic cysts.

## Imaging features of cystic components

In brain neoplasm imaging, distinguishing true cysts from necrotic changes can provide useful pathological insight, although this distinction can occasionally be challenging. True cysts are typically characterized by well-circumscribed, rounded areas that demonstrate signal intensities similar to cerebrospinal fluid, with very high signal on T2-weighted images and low signal on T1-weighted images. They usually have thin, smooth walls that may show no enhancement or only minimal, regular enhancement, and occasionally contain fine internal septations [7]. They often show high apparent diffusion coefficient (ADC) values on diffusion-weighted imaging (DWI) [8]. In contrast, necrotic cavities, which are often observed in high-grade tumors, represent non-viable tumor tissue and are generally seen as regions with absent or markedly reduced enhancement within the tumor. These areas often appear hyperintense on T2-weighted images and hypointense on T1-weighted images, but unlike true cysts, they tend to have irregular, ill-defined margins rather than a smooth, well-defined wall [7]. Necrotic cavities may show greater heterogeneity of ADC values due to internal debris, hemorrhage, or proteinaceous material [8]. In this review, the term ‘cystic component’ is used to refer to fluid-containing regions within tumors on imaging, including true cyst and necrotic cavities.

## Role of advanced MRI in the diagnosis of cystic brain neoplasms

Various advanced MRI techniques, including DWI, perfusion-weighted imaging (PWI), and MR spectroscopy (MRS), provide complementary information that can improve the characterization of both solid and cystic components in brain neoplasms.

DWI reflects tissue cellularity and is useful for differentiating cystic tumor components from abscesses. While cystic components typically show elevated ADC values, abscesses characteristically demonstrate marked diffusion restriction due to highly viscous purulent material. Lower ADC values may also be associated with higher-grade tumors [9].

PWI provides information on tumor vascularity and angiogenesis. In general, enhancing components of high-grade tumors demonstrate elevated relative cerebral blood volume (rCBV), whereas low-grade tumors tend to show lower perfusion [10]. Elevated rCBV may also aid in distinguishing high-grade tumors from non-neoplastic lesions such as abscesses or radiation necrosis [11]. Perfusion characteristics vary among tumor types, reflecting differences in vascularity, and can aid in differential diagnosis [12, 13].

MRS provides metabolic information, with high-grade tumors often showing elevated choline and lipid/lactate peaks associated with increased membrane turnover and necrosis or hypoxia [14].

These techniques should be interpreted in conjunction with conventional MRI findings, and a multiparametric approach may improve diagnostic confidence.

## Artificial intelligence for cystic brain neoplasms

Beyond conventional imaging features, artificial intelligence (AI) and radiomics are increasingly being explored. These techniques enable quantitative characterization of brain tumors and have shown potential in neuroradiology [15–21]. Radiomics allows the extraction of high-dimensional quantitative data from MRI scans, capturing subtle differences in signal intensity, texture, and spatial relationships between cystic and solid components. When integrated with AI-based classification models, these approaches have shown potential utility in challenging differential diagnoses, such as distinction of cystic neoplasms from brain abscesses, PA from cystic oligodendroglioma, and two molecular subtypes of ependymoma [22–25]. At present, however, these AI-based methods remain investigational and should be regarded as complementary tools rather than established clinical standards, pending further multi-center validation.

## Hemangioblastoma

HB is a benign, vascular neoplasm categorized as a World Health Organization (WHO) grade 1 tumor [26]. HB is the most frequent primary neoplasm of the posterior fossa in adults [27]. The cerebellum is the most common site of origin, although lesions can also occur in the brainstem, spinal cord, optic nerve, and retina [28]. Despite being a benign neoplasm, it can lead to serious neurological deficits by causing hydrocephalus, herniation, or brainstem compression, and may occasionally result in intracranial hemorrhage. HBs are histologically classified into two subtypes: the reticular and cellular types. The majority of HBs fall into the reticular category, characterized by a rich network of capillaries interspersed with stromal cells. In contrast, the cellular type is less common and consists predominantly of densely packed stromal cells with relatively fewer vascular components [29].

Approximately a quarter of cases are linked to von Hippel-Lindau (VHL) disease, in which patients tend to present with multiple lesions and an earlier age of onset compared to sporadic cases. Sixty–eighty percent of patients with VHL develop CNS HBs [4, 30], and in about 40% of cases, these tumors represent the initial clinical manifestation [30, 31]. HBs also account for approximately half of all VHL-related deaths [31]. Given their prevalence and clinical impact, routine screening for CNS HBs using neurological assessment and MRI is recommended in patients with VHL [31].

**Fig. 1:**
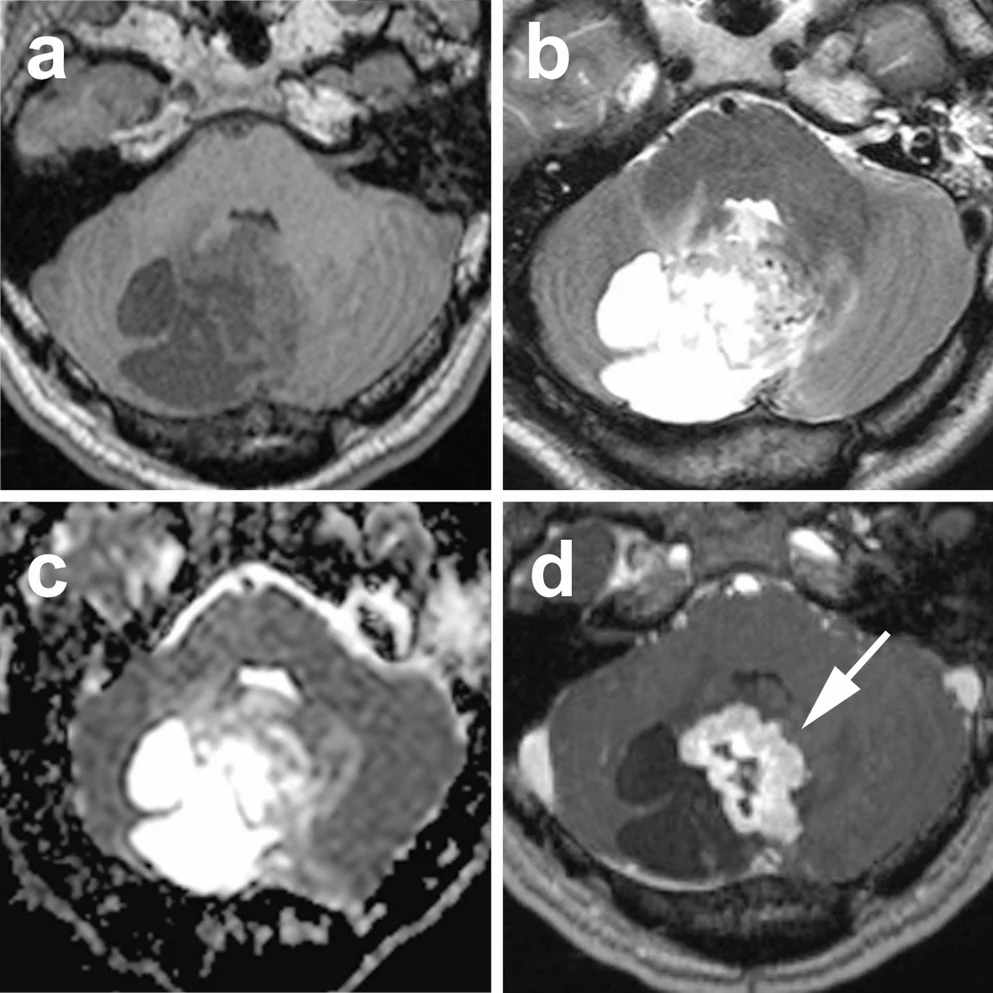
MR images of a 56-year-old man with hemangioblastoma presenting with visual disturbance and headache. Axial T1-weighted image (**a**), T2-weighted image (**b**), apparent diffusion coefficient map (**c**), and contrast-enhanced T1-weighted image (**d**). The lesion demonstrates a multilocular cystic mass with a relatively large, irregular solid component. The solid portion shows high signal intensity on T2-weighted images with flow voids, indicating prominent vascularity, and exhibits marked enhancement after contrast administration (arrow) without evidence of restricted diffusion

On imaging, HBs often present a large cyst with an enhancing mural nodule (Fig. 1), but some cases present as solid tumors without associated cystic components [32]. They are thought to initially appear as solid tumors with peritumoral edema, with the cyst subsequently developing from progressive accumulation of edema fluid secondary to increased vascular permeability [3, 4]. The cystic component is typically hyperintense on T2-weighted MR images and hypointense on T1-weighted images. The mural nodule demonstrates intense contrast enhancement and may show flow voids on T2-weighted images, reflecting its rich vascularity. One notable feature is the lack of enhancement in the cyst wall, helping differentiate HB from other cystic tumors such as PA, which may show variable wall enhancement. On CT imaging, the cyst is hypodense, and the solid component may appear isodense or slightly hyperdense. Hemorrhage and calcification are uncommon. Additionally, HBs associated with VHL disease have been shown to express somatostatin receptors (SSTR). Therefore, SSTR-targeted imaging such as ^68^ Ga-DOTATATE and ^68^ Ga-DOTATOC PET/CT can demonstrate uptake and could serve as a complementary modality for whole-body surveillance in VHL patients [33].

PA may mimic the imaging appearance of HB, especially in younger patients. Metastatic lesions, particularly from hypervascular primaries such as renal cell carcinoma and thyroid carcinoma, may also resemble HB. Advanced imaging methods such as DWI and PWI may help diagnosing HB. This tumor has been shown to exhibit significantly higher ADC value compared to metastatic tumors [34]. Several studies have demonstrated the relatively high vascularity of HBs based on perfusion metrics. Compared to metastatic tumors, HBs show significantly elevated rCBV [34]. When compared with PAs and medulloblastomas, HBs exhibit higher normalized relative cerebral blood flow (nrCBF) [13]. In addition, both rCBV and relative peak height (rPH) on PWI are significantly higher in HBs than in PAs [12, 35].

Surgical excision remains the primary treatment and is usually curative in sporadic cases. Preoperative embolization may be considered in larger or highly vascular tumors to reduce intraoperative bleeding. In the context of VHL disease, long-term surveillance is recommended due to the potential for recurrence or development of new lesions [31].

## Pilocytic astrocytoma

PA is a slow-growing glial tumor classified as WHO grade 1. It is the most common pediatric glioma and typically affects children and adolescents, with two-thirds of cases diagnosed before the age of 18 [36]. This tumor most frequently arises in the cerebellum, followed by the optic chiasm, hypothalamus, and brainstem. Involvement of less common sites such as the cerebral hemispheres, ventricular compartments and spinal cord has also been reported [37–39]. In adults, supratentorial locations such as the frontal lobes are more frequently encountered than in children. PA demonstrates a strong association with neurofibromatosis type 1 (NF1). Optic pathway gliomas occur in approximately 15–25% of individuals with NF1 [40], and are most commonly diagnosed before the age of 6. A female predominance has been observed, with a female-to-male ratio of approximately 2:1 [41]. Histologically, PA typically exhibits a biphasic pattern, with compacted piloid regions and loose glial areas. The piloid component contains elongated bipolar cells with fine fibrillary processes and abundant Rosenthal fibers. In contrast, the loose areas often show microcysts, vacuoles, and multipolar astrocytes associated with eosinophilic granular bodies [36].

**Fig. 2:**
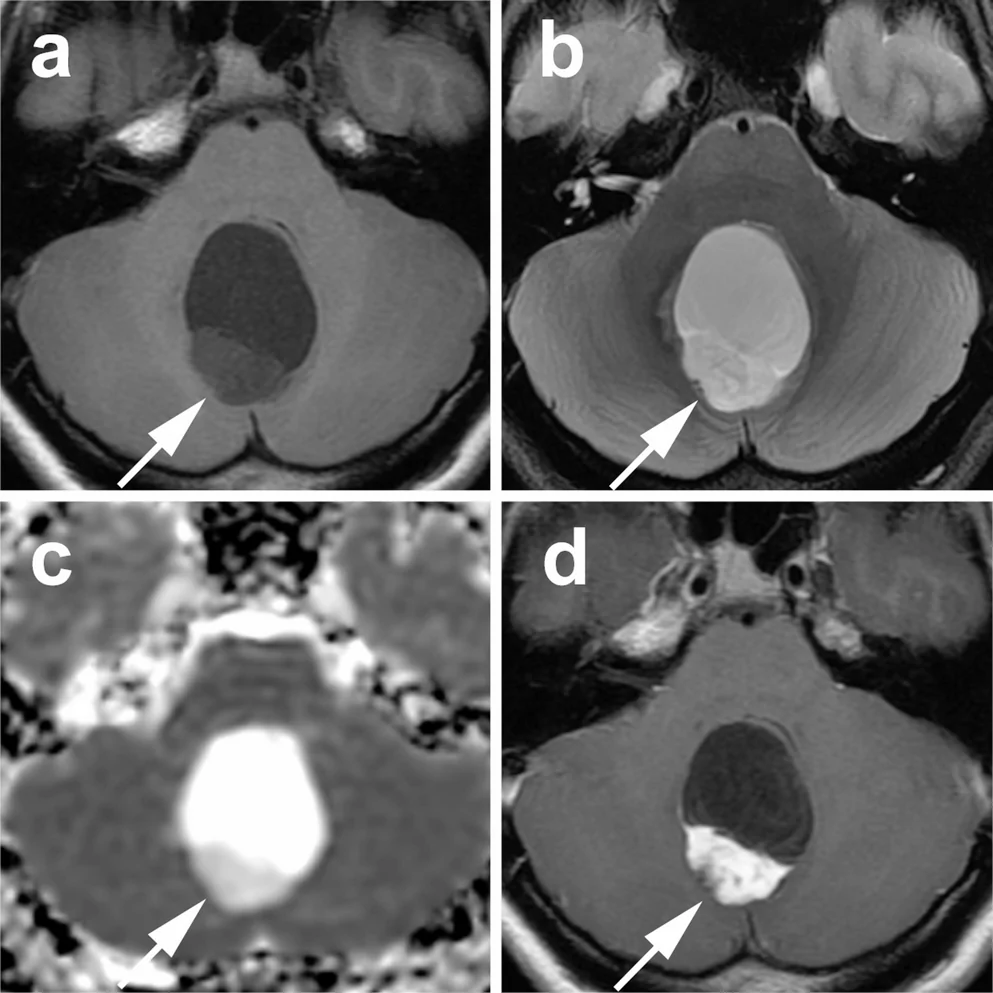
A 19-year-old woman with pilocytic astrocytoma presenting with dizziness, gait disturbance, headache, and vomiting. Axial MR images demonstrate a cystic component with a dorsally located solid portion (arrows) that exhibits low signal intensity on T1-weighted images (**a**), high signal intensity on T2-weighted images (**b**), high ADC values (**c**), and contrast enhancement (**d**). The fourth ventricle is compressed by the tumor

On MRI, the classic cerebellar form appears as a large cyst with a mural nodule (Fig. 2). The cyst is typically hyperintense on T2-weighted images and hypointense on T1-weighted images, while the solid component enhances strongly after gadolinium administration [36]. Tumors involving the optic pathway or hypothalamus often exhibit a more solid appearance. The cyst wall may show enhancement in approximately half of cases. Calcification and hemorrhage have been reported but are uncommon. In previous studies, PAs showed significantly higher ADC values on DWI than medulloblastomas and ependymomas [13, 42], and lower rCBV or nrCBF on PWI than HBs [12, 13], reflecting their relatively low cellularity and vascularity. Although it is classified as Grade 1 neoplasm, it can occasionally demonstrate local recurrence or malignant transformation with dissemination [36, 43].

Surgical resection is usually curative in cerebellar and hemispheric lesions. Tumors involving deep midline structures, such as the optic pathway, may be managed conservatively or with adjuvant therapies depending on location and progression. Recurrence is rare after gross total resection, and overall prognosis is excellent.

## Pleomorphic xanthoastrocytoma

PXA is an uncommon glial tumor, classified as WHO grade 2 or, when exhibiting anaplastic features, grade 3. It typically affects children and young adults, with a peak incidence in the second decade of life. The most frequent location is the superficial cerebral cortex, especially in the temporal lobes, though frontal and parietal involvement also occurs [44]. Seizures are a common clinical presentation due to the cortical location [45]. Histologically, PXA is characterized by marked cellular pleomorphism, the presence of lipid-laden xanthomatous astrocytes, and a reticulin-rich background. Despite the prominent nuclear atypia, the mitotic activity is generally low in grade 2 tumors.

**Fig. 3:**
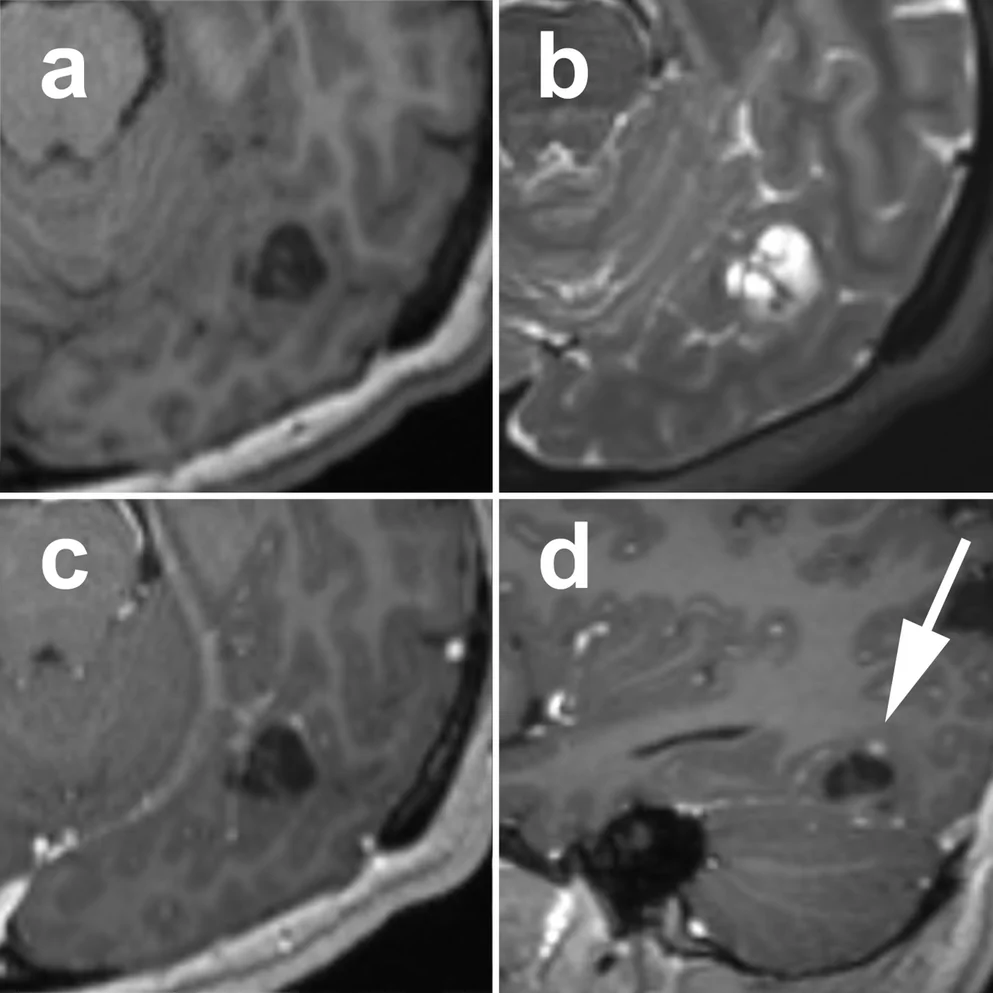
A 21-year-old woman with pleomorphic xanthoastrocytoma presenting with seizures. Axial T1-weighted image (**a**), axial T2-weighted image (**b**), axial and sagittal contrast-enhanced T1-weighted images (c and d, respectively). A cystic lesion with an enhancing mural nodule (arrow) is seen in the cortical region of the posterior lobe. No peritumoral edema is observed

On imaging, PXAs often appear as well-circumscribed, cortically-based cystic masses with an enhancing mural nodule (Fig. 3), but this neoplasm can also present without cystic component [46]. The cyst wall and solid component may enhance with contrast, though the pattern is variable. The solid portion typically shows hypointense signal on T1-weighted images and mildly hyperintense signal on T2-weighted images. Hemorrhage and calcification can occur but are not common. Peritumoral edema is typically absent or minimal in PXAs, a feature that may help distinguish it from high-grade gliomas. Remodeling of the inner table of the skull has been reported, which is a nonspecific imaging finding of superficial brain tumors of childhood including ganglioglioma and DNET [47]. Additionally, PXAs can demonstrate leptomeningeal enhancement, which is one of their characteristic imaging features [46, 48]. CSF dissemination is rare but may be seen in anaplastic variants. Increased FDG uptake on PET has been reported in some cases, such as recurrent PXA [49, 50].

Surgical resection is the primary treatment. Gross total resection is associated with a favorable prognosis, especially for grade 2 tumors. Anaplastic transformation or recurrence may warrant adjuvant radiotherapy or chemotherapy [44].

## Ganglioglioma

Ganglioglioma is a rare, slow-growing neoplasm composed of a mixture of neoplastic ganglion cells and glial elements. It is classified as WHO grade 1 and accounts for less than 1% of intracranial tumors. The diagnosis of anaplastic ganglioglioma is currently controversial and the 2021 WHO classification no longer recognizes it as a distinct diagnostic entity [26]. Previously, this term was applied to tumors exhibiting features such as increased mitotic activity, microvascular proliferation, or necrosis, but many previously reported cases may have lacked adequate molecular testing to exclude other high-grade gliomas [26, 51, 52]. Gangliogliomas most frequently arise in children and young adults, and are most commonly located in the temporal lobe cortex, although they can also occur in other parts of the cerebral hemispheres, brainstem, or cerebellum [46, 53]. Patients typically present with seizures, often refractory to medication, particularly when the tumor is located in the mesial temporal lobe [45]. Some gangliogliomas are associated with focal cortical dysplasia, especially in cases presenting with epilepsy.

**Fig. 4:**
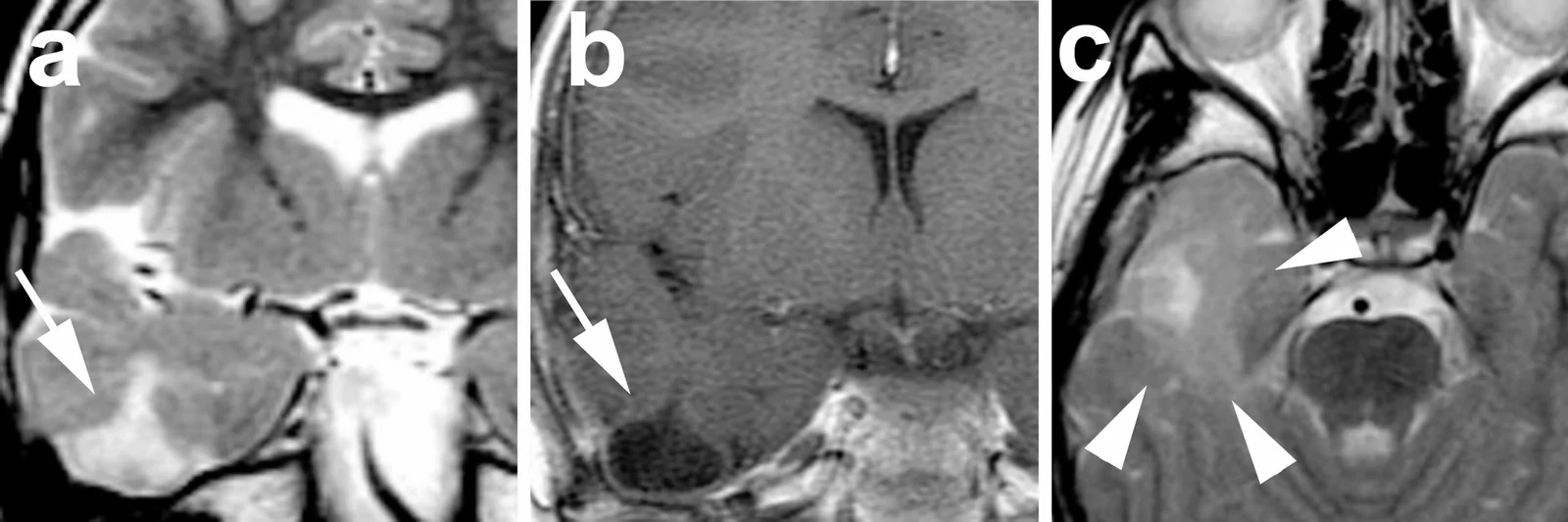
A 7-year-old girl with a ganglioglioma presenting with facial spasms and left arm automatisms. Coronal T2-weighted image (**a**) and contrast-enhanced coronal T1-weighted image (**b**) show a cystic mass in the right temporal cortex without appreciable contrast enhancement (arrows). On the axial T2-weighted image (**c**), ill-defined hyperintensity is observed in the adjacent cortex and white matter (arrowheads), corresponding to focal cortical dysplasia associated with the tumor

Imaging characteristics are variable but commonly include well-circumscribed lesions with both cystic and solid components [46, 53, 54] (Fig. 4). In a large series, the majority of tumors were found to be mixed cystic-solid (approximately 40–50%), while purely solid and purely cystic forms were less common. The solid component is usually hypointense to isointense on T1-weighted images and hyperintense on T2-weighted images. Calcification is observed in up to one-third of cases and is more easily detected by CT. Contrast enhancement is highly variable, ranging from absent to minimal, ring-like, or diffuse enhancement. In some cases, no enhancement is observed, which can make diagnosis particularly challenging. Peritumoral edema is usually absent or minimal. Scalloping or thinning of the adjacent calvarium may be visible, indicating chronic pressure on the bone. Posterior fossa gangliogliomas tend to be larger and more solid than supratentorial ones and may occasionally lead to ipsilateral cerebellar cortical atrophy [55].

Surgical resection is generally curative, especially for tumors causing seizures. The prognosis is favorable following complete excision, though anaplastic transformation is a rare complication requiring close follow-up.

## Desmoplastic infantile ganglioglioma/astrocytoma

DIG and DIA are rare, WHO grade 1 pediatric neoplasm that predominantly affect infants under two years of age. DIGs contain both neuronal and glial components, whereas DIAs are composed purely of glial cells. One of the most frequent symptoms is a rapid enlargement of head circumference. Seizure is uncommon. Despite their aggressive appearance on imaging, these tumors have a favorable prognosis after resection [46].

Imaging reveals a large, often massive lesion with a dominant cystic component and a mural nodule located superficially near the cortex of the frontal and parietal lobes. Less frequently, lesions in the temporal and occipital lobes have been reported. The solid component may appear hyperdense on CT. T1-weighted MRI shows the solid nodule as iso- to hypointense with intense contrast enhancement [46, 53, 56]. The mural nodule frequently lies close to the dura mater, and a dural tail-like enhancement may be seen. Calcification is uncommon. Despite their size, peritumoral edema and mass effect are often minimal, and the lesion grows expansively rather than infiltratively. The differential diagnosis includes other cystic neoplasms of infants; however, the relative rarity of calcification may aid in distinguishing this entity from neoplasms that frequently calcify within the first year of life, such as embryonal tumors and ependymoma [46]. The age at presentation, cortical-based location, and cystic morphology with a dural-based solid nodule also support the diagnosis of DIG/DIA.

Although surgical resection is typically curative, complete resection is often challenging due to the large size of these tumors and their firm dural attachment [46]. In cases of subtotal resection, careful follow-up is essential, and if recurrence or progression is observed and further surgery is not feasible, chemotherapy may be considered as an alternative treatment option [57].

## Dysembryoplastic neuroepithelial tumor

DNET is a benign, WHO grade 1 tumor characterized by a distinct glioneuronal composition. It typically presents in childhood or adolescence, with a slight male predominance, and is strongly associated with treatment-refractory epilepsy [45]. Most tumors arise in the cortical gray matter, particularly in the temporal lobes, though frontal and parietal involvement is also frequent [58–60]. Although DNETs are predominantly cortical-based tumors, rare cases have been described in the septal region or within the ventricular system [61, 62]. Multifocal presentation is uncommon but has been reported [63].

DNETs are histologically characterized by oligodendrocyte-like cells embedded in a mucin-rich matrix, forming a distinctive structure known as the “specific glioneuronal element.” DNETs may be associated with focal cortical dysplasia or hippocampal sclerosis, particularly in cases presenting with epilepsy [58]. Although three histologic subtypes of DNET, referred to as complex, simple, and non-specific, were previously described, this classification is no longer included in the current WHO criteria [26, 58, 64]. DNETs are frequently associated with genetic alterations in the FGFR1 gene, most notably duplications of its tyrosine kinase domain [26, 65].

**Fig. 5:**
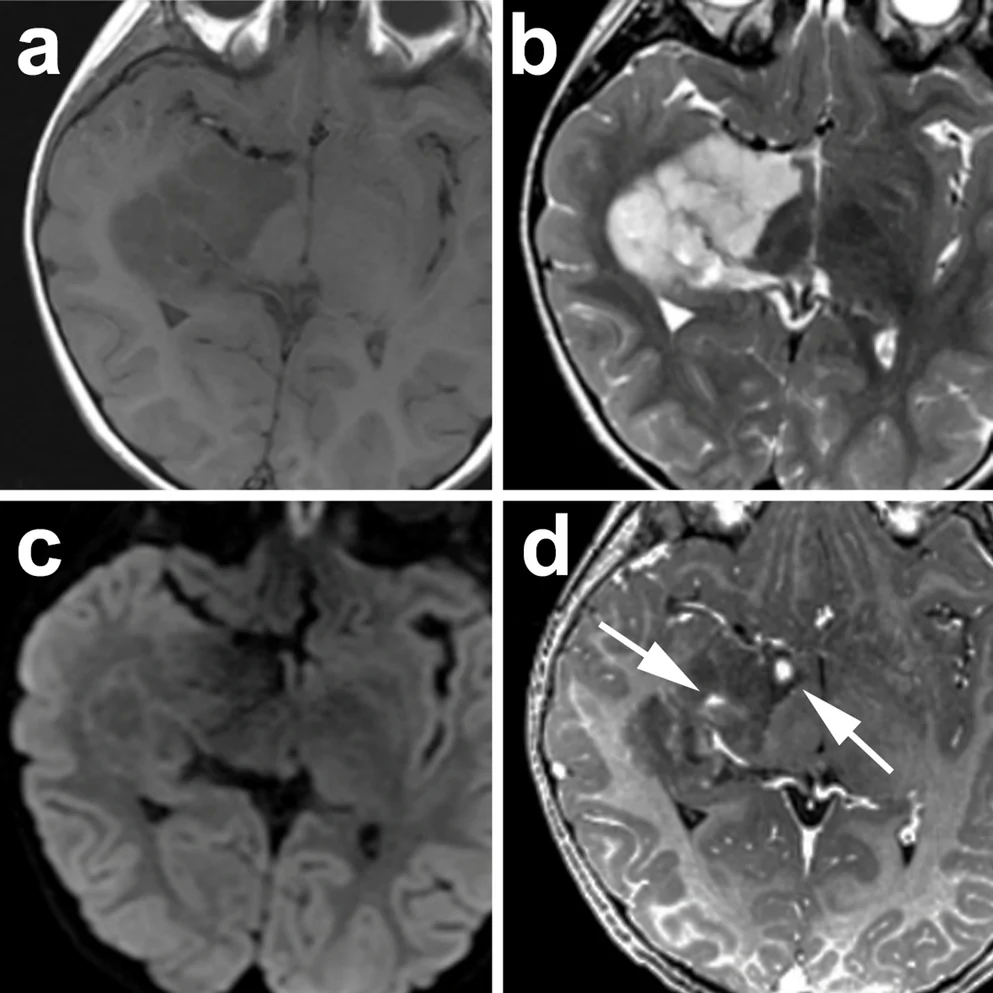
A 4-year-old boy with dysembryoplastic neuroepithelial tumor presenting with absence seizures. A well-circumscribed mass with a cystic-like appearance is seen in the right mesial temporal lobe, showing low signal intensity on the T1-weighted image (**a**) and high signal intensity on the T2-weighted image (**b**). No obvious hyperintensity is observed on the diffusion-weighted images (**c**). The contrast-enhanced T1-weighted image (**d**) demonstrates nodular enhancement (arrows)

MRI findings include a well-demarcated cortical lesion with a “bubbly” or multicystic appearance on T2-weighted images [46, 53, 59, 60, 66] (Fig. 5). The lesion is usually hypointense on T1-weighted and hyperintense on T2-weighted images. FLAIR may show a hyperintense rim or a multiloculated pattern. High ADC values are common, reflecting the tumor’s low cellularity. Contrast enhancement is variable but often mild or absent. On PWI, rCBV is typically low. When present, enhancement may be nodular or ring-like. Calcification is observed in up to 30% of cases. Mass effect and vasogenic edema are typically minimal, and cortical expansion rather than infiltration is the dominant growth pattern. Adjacent bone remodeling is often observed [46, 53]. The differential diagnosis includes low-grade gliomas and ganglioglioma. Features such as absence of edema or enhancement and stability over time may be suggestive of DNET.

Surgical resection usually leads to good seizure control outcomes. Complete excision typically results in a low recurrence rate, and long-term prognosis is excellent. Because of its benign nature, adjuvant therapy is not indicated. However, rare cases of malignant transformation have been reported. Among these cases, 65% arose outside the temporal lobe and 93% demonstrated contrast enhancement on post-contrast T1-weighted images [67].

## Central neurocytoma

Central neurocytoma is a typically benign neoplasm, classified as WHO grade 2 [68]. It constitutes less than 1% of intracranial neoplasms and most frequently affects young adults between 20 and 40 years of age [68, 69]. The tumor originates from the lateral walls of the lateral ventricles, near the foramen of Monro or septum pellucidum, and occasionally extends into the third ventricle [70]. Extraventricular neurocytomas have also been reported, with occurrences in the brain parenchyma, cerebellum, and spinal cord. Symptoms often include headache, nausea, or other signs of increased intracranial pressure. Histologically, it is composed of uniform round cells with neuronal differentiation. Immunohistochemistry typically reveals positivity for neuronal markers such as synaptophysin. Although the precise histogenetic origin of these tumors remains uncertain, in vitro studies indicate that they may derive from bipotential precursor cells with the capacity to differentiate into both neuronal and glial lineages [68].

**Fig. 6:**
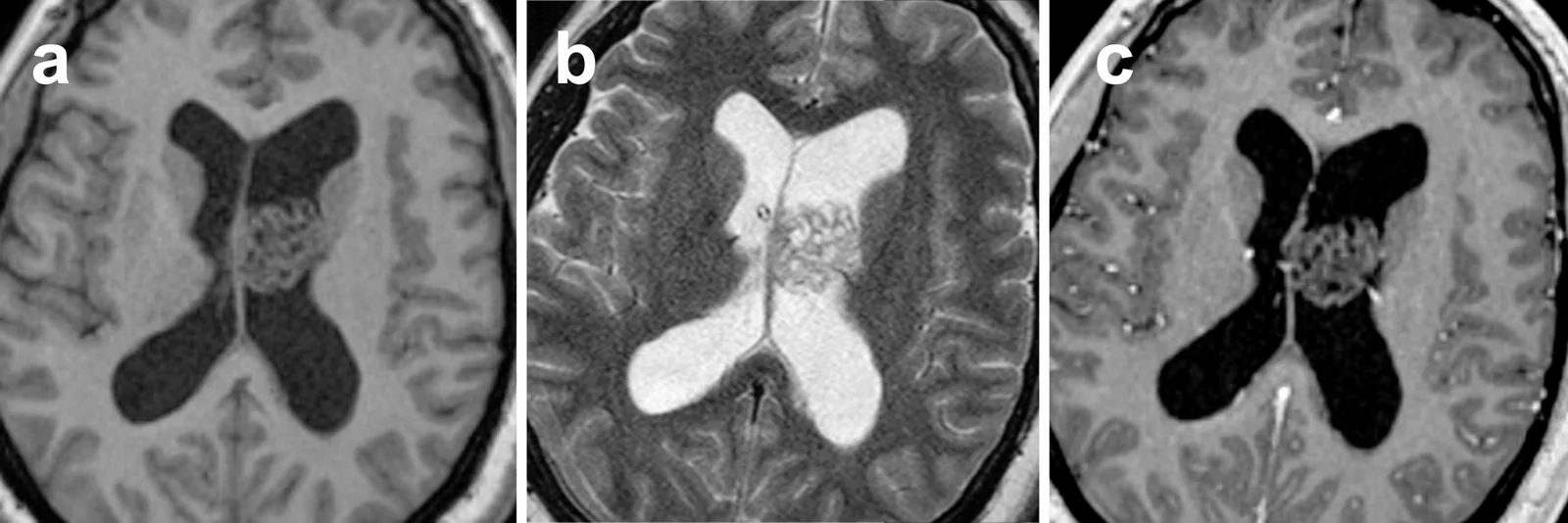
A 24-year-old man with central neurocytoma presenting with headache. Axial T1-weighted image (**a**), T2-weighted image (**b**), and contrast-enhanced T1-weighted image (c). A multilobulated “soap-bubble-like” tumor is seen near the foramen of Monro, composed of multiple cystic areas and showing minimal contrast enhancement

The imaging characteristics typically include a well-defined intraventricular mass with mixed solid and cystic components (Fig. 6). The classic “soap-bubble” appearance arises from multiple small cystic spaces within the solid tumor matrix [68, 70, 71]. Cystic components are frequently located at the periphery of the tumor [70]. Moreover, the scalloping sign, which refers to undulations or spicules along the lateral ventricular wall adjacent to the tumor capsule, may be a useful imaging feature for distinguishing central neurocytoma from other intraventricular tumors [70, 72]. On T1-weighted images, the tumor appears iso- to hypointense, while T2-weighted images show heterogeneous hyperintensity. Enhancement after gadolinium administration is typically moderate and heterogeneous [14]. Calcifications are present in approximately 50% of cases, and hemorrhage, though rare, may occasionally be observed [53, 68]. DWI shows variable restriction. Lower minimum ADC values on DWI have been reported to correlate with higher proliferative activity [73]. MRS often reveals a decreased NAA peak, a moderately elevated Cho peak, and the presence of glycine and alanine peaks [14]. Obstructive hydrocephalus may be caused by compression of the foramen of Monro. Differential diagnosis includes intraventricular ependymoma, subependymoma, and oligodendroglioma.

Surgical resection is the primary treatment, and gross total resection is associated with favorable prognosis, although recurrence and CSF dissemination have been reported [74, 75]. In cases of subtotal resection or atypical histologic features, adjuvant radiotherapy may be considered. Long-term survival is excellent with appropriate management.

## Ependymoma

Ependymoma is a glial tumor derived from ependymal cells lining the ventricular system and the central canal of the spinal cord and is classified as grade 2 or 3 [26]. They account for approximately 3% to 5% of all intracranial tumors and arise either in the supratentorial region (approximately 40% of cases) or within the posterior fossa (around 60%) [68].

The 2021 WHO classification categorizes ependymal tumors according to their histopathological, molecular, and anatomical characteristics, grouping them into supratentorial, posterior fossa, and spinal types [76]. Supratentorial ependymomas are further classified into two molecular subtypes based on gene fusions: ZFTA fusion-positive and YAP1 fusion-positive. Posterior fossa ependymomas are divided into two groups, PFA and PFB, according to methylation profiling. In the spinal area, a newly recognized subgroup defined by MYCN amplification has been associated with more aggressive clinical behavior. Histopathologically, ependymomas display features such as perivascular pseudorosettes and ependymal rosettes, reflecting their origin.

**Fig. 7:**
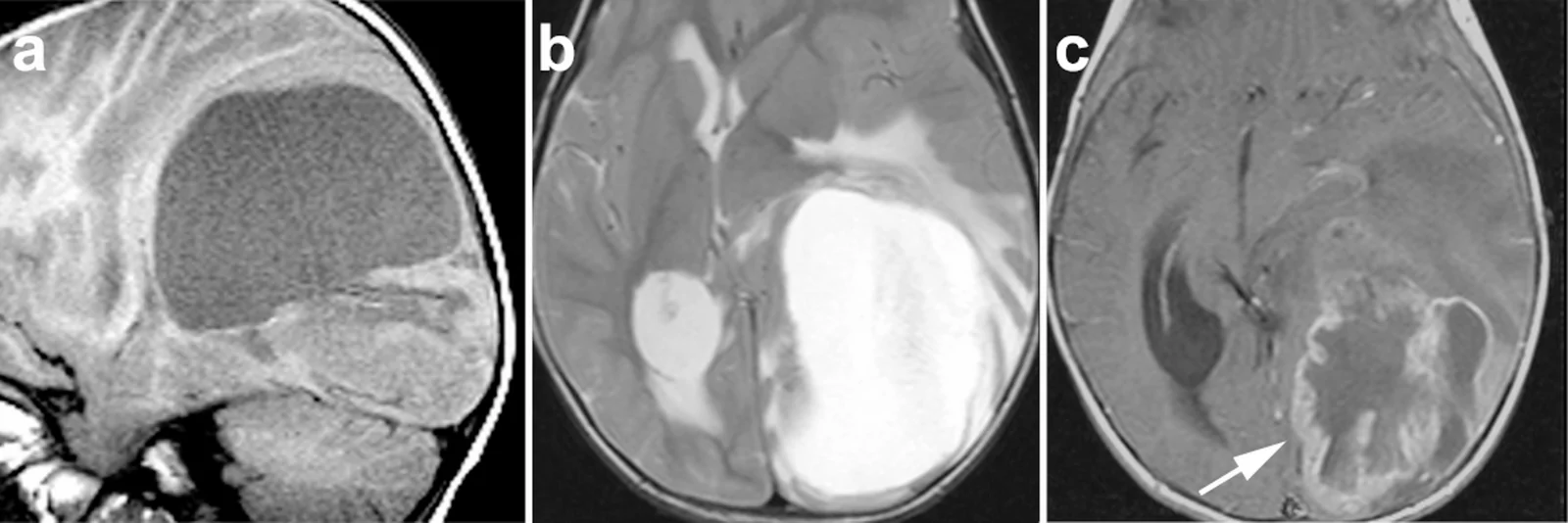
A case of supratentorial ependymoma. Sagittal T1-weighted image (**a**), axial T2-weighted image (**b**), and contrast-enhanced axial T1-weighted image (**c**). A large cystic lesion is seen in the left occipital lobe with peripheral enhancement (arrow). Peritumoral edema is present, accompanied by a midline shift to the right

Supratentorial ependymomas primarily occur in the pediatric and adolescent populations. ZFTA and YAP1 markers are commonly found in pediatric patients. The average age at diagnosis is approximately 8 years for ZFTA fusion-positive tumors, whereas YAP1 fusion-positive tumors tend to present much earlier, with a mean age of around 1.4 years [77]. YAP1 fusion-positive ependymomas are less common than ZFTA fusion-positive tumors, and they tend to occur slightly more often in female patients. Clinical presentation includes headache, seizures, or focal neurological symptoms. ZFTA fusion-positive supratentorial ependymomas are associated with a poorer prognosis, with 10-year overall survival rates reported around 50% [77]. In contrast, YAP1 fusion-positive tumors demonstrate a more favorable clinical course, with 10-year survival ranging from 88 to 100% [78]. In adults, supratentorial ependymomas tend to originate along the ventricular lining, while in children they are more often located within the parenchyma without direct ventricular involvement (Fig. 7). Heterogeneous contrast enhancement and restricted diffusion within the solid component are often observed. Necrosis, calcification, and hemorrhage are common. The degree of vasogenic edema surrounding the tumor is variable [68, 71, 76, 79, 80].

**Fig. 8:**
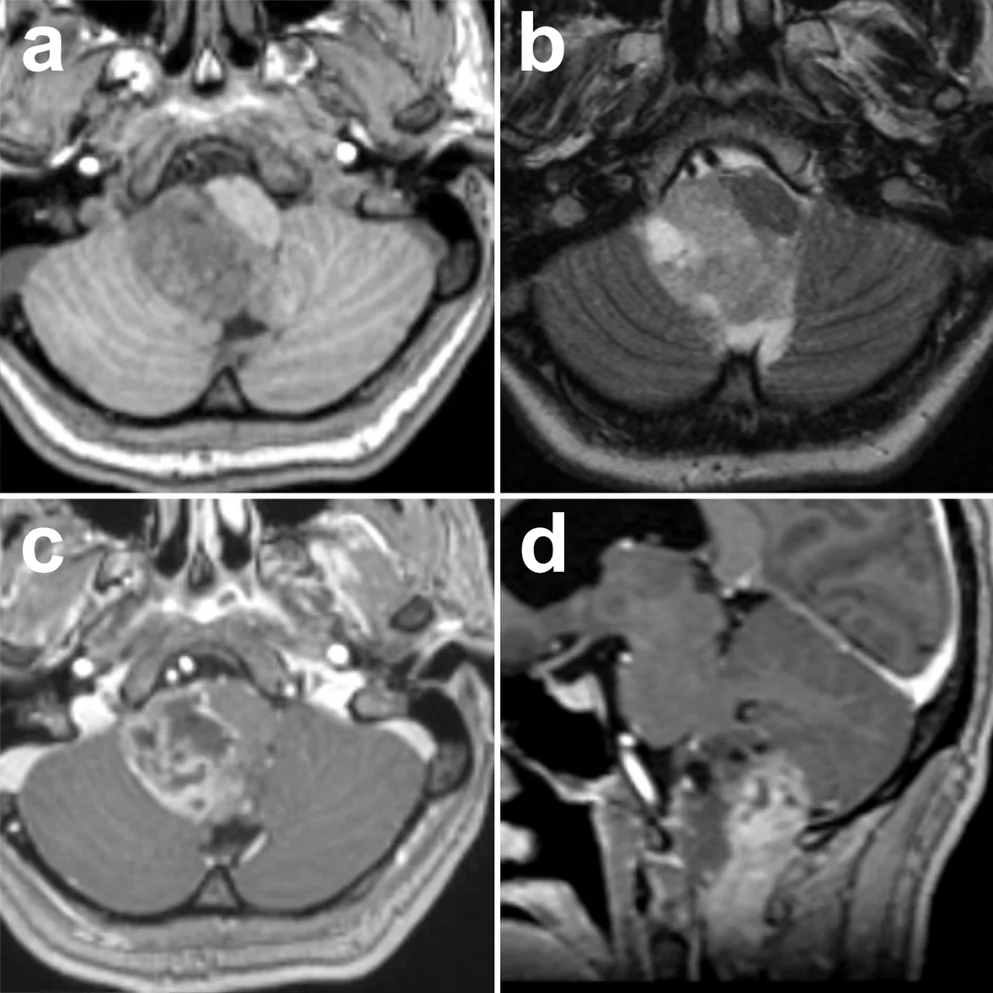
A 37-year-old woman with posterior fossa ependymoma, molecular subtype PFB, presenting with headache. Axial T1-weighted image (**a**), axial T2-weighted image (**b**), and contrast-enhanced axial and sagittal T1-weighted images (**c** and **d**, respectively). The tumor is centered in the right foramen of Luschka and caudally through the foramen magnum to the C3 level of the spinal canal. It consists of mixed solid and cystic components with heterogeneous enhancement. No apparent hemorrhage or calcification is observed. The medulla is compressed, resulting in obstructive hydrocephalus

Posterior fossa ependymomas are usually seen in children, with a mean age of presentation around six years [68]. They commonly present with signs of elevated intracranial pressure and cerebellar dysfunction. Among the two molecular subtypes, PFA ependymomas are more frequently diagnosed in infants and young children, with a mean age of around 3 years and a slight male predominance. They are associated with a less favorable prognosis, showing a 10-year overall survival rate of approximately 56%, and they have a higher propensity for recurrence and dissemination [77, 81]. In contrast, PFB ependymomas typically occur in adolescents and young adults, with a mean age near 30 years and a slight female predominance. Prognosis for PFB tumors is generally favorable, with reported 10-year survival rates approaching 88%. PFA ependymomas are relatively large, often exhibit calcifications, and tend to arise in midline or lateral locations. They tend to involve the roof or lateral walls of the fourth ventricle and may extend into the foramen of Luschka, frequently resulting in hydrocephalus. In contrast, PFB ependymomas are generally smaller, non-calcified, and less invasive, typically arising from the midline floor of the fourth ventricle. Compared to PFA tumors, PFB ependymomas more often show cyst formation and homogeneous pattern of contrast enhancement [81, 82] (Fig. 8). While medulloblastomas, an important differential diagnosis in pediatric posterior fossa tumors, typically demonstrates marked diffusion restriction, ependymomas tend to show relatively less pronounced ADC reduction [76]. However, ADC interpretation may be limited in the presence of calcification or hemorrhage.

Treatment is surgical resection, with the extent of resection being a critical prognostic factor. Adjuvant radiation therapy is often recommended for incompletely resected or high-grade lesions. Long-term follow-up is required due to the risk of recurrence and late dissemination.

## Glioblastoma

Glioblastoma, classified as WHO grade 4, is the most aggressive primary malignant brain tumor in adults and accounts for approximately 16% of all intracranial neoplasms [83]. Symptoms are nonspecific and include headache, focal neurological deficits, or seizures. The diagnosis of glioblastoma was previously based on histopathological criteria, but molecular markers have become increasingly important in recent classifications [26, 84, 85]. The 2021 WHO classification defines glioblastoma as “diffuse, astrocytic glioma that is IDH-wild-type and H3-wild-type and has one or more of the following histologic or genetic features: microvascular proliferation, necrosis, TERT promoter mutation, EGFR gene amplification, + 7/ − 10 chromosome copy-number changes (CNS WHO grade 4)” [26, 86]. From a radiogenomic perspective, while glioblastomas are typically characterized by necrosis, heterogeneous contrast enhancement, and increased perfusion, those defined by molecular criteria may present as infiltrative, non-enhancing or only faintly enhancing lesions with minimal necrosis, mimicking lower-grade gliomas [87].

Glioblastomas may contain cystic components on imaging. It should be noted that these fluid-like components often represent irregular necrotic degeneration. In contrast, a subset of cases demonstrates true cystic components, which may be distinguished from necrosis by relatively smooth, thin margins of enhancement surrounding the non-enhancing fluid that is isointense to cerebrospinal fluid. Approximately 8–10% of glioblastoma cases exhibit prominent cystic components [88, 89]. A report comparing cystic (defined as having cystic components distinct from necrosis) and non-cystic glioblastomas has indicated that the prevalence of IDH1 mutation and MGMT promoter methylation does not differ significantly between the two groups [88]. Although several studies have reported improved survival in patients with cystic glioblastoma, these findings are inconsistent and often derived from cohorts in which molecular characteristics, including IDH mutation status, were not adequately controlled for [6, 89–95]. At present, no definitive conclusions can be drawn regarding the prognostic impact of cystic components.

**Fig. 9:**
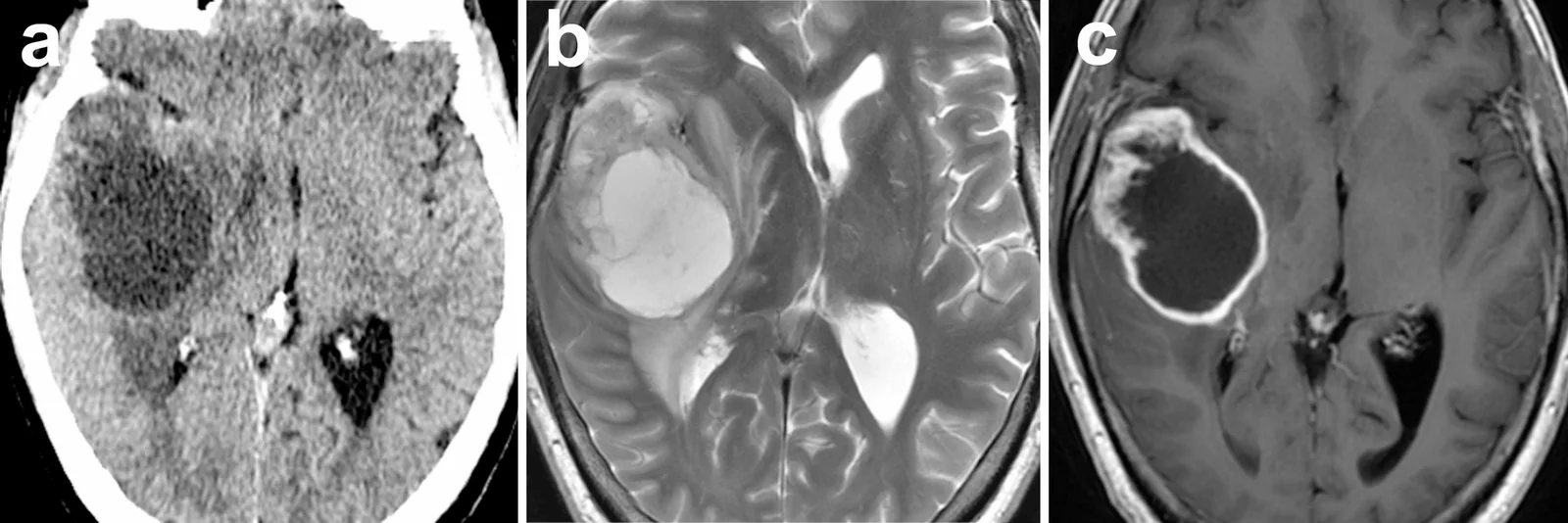
A 65-year-old man with glioblastoma presenting with headache. CT image (**a**) shows a cystic lesion in the right temporal lobe with surrounding edema and a midline shift to the left. On MRI, the T2-weighted image (**b**) demonstrates the cystic lesion, and the contrast-enhanced T1-weighted image (c) reveals irregular thick enhancement along the cyst wall

On MRI, the cystic component is hyperintense on T2-weighted images and hypointense on T1-weighted sequences. The solid component shows heterogeneous enhancement after gadolinium administration (Fig. 9). Peritumoral vasogenic edema is typically extensive. Diffusion restriction is often observed in the solid component, whereas the cystic component typically shows elevated ADC values, although intralesional debris or hemorrhage may occasionally alter this pattern when the fluid-containing component represents necrosis. An important differential diagnosis for cystic lesions with rim enhancement is brain abscess; however, abscesses typically show marked hyperintensity within the cavity on DWI, which may aid in differentiation from cystic glioblastoma. On PWI, elevated rCBV is typically observed in enhancing tumor portions, which has been suggested to be useful for differentiating glioblastoma from other lesions such as abscess and radiation necrosis. MRS may demonstrate elevated choline and lipid/lactate peaks, consistent with high-grade malignancy. Cystic glioblastomas may pose diagnostic challenges due to overlap with less aggressive cystic lesions such as gangliogliomas or PAs [96].

The initial treatment is maximal safe surgical resection followed by radiotherapy and temozolomide chemotherapy. Despite multimodal therapy, prognosis remains poor, with median survival of approximately 15 months [97]. To ensure consistency in treatment response evaluation, the updated Response Assessment in Neuro-Oncology (RANO) criteria version 2.0 recommends a standard set of criteria for both high- and low-grade gliomas and contrast-enhancing or non-contrast-enhancing lesions with clearly defined margins by MRI scan. Measurement of tumor around a cyst or surgical cavity remains challenging; such lesions are generally considered nonmeasurable unless there is a nodular enhancing component measuring ≥ 10 mm in diameter. The cystic or surgical cavity itself should not be measured when determining therapeutic response [18, 21].

## Brain metastasis

While solid lesions are more common for metastatic brain neoplasms, cystic-appearing lesions may occur in up to 10–20%. Primary origins of cystic brain metastases include lung carcinoma, breast cancer, renal cell carcinoma, melanoma and thymic carcinoma [98–104].

**Fig. 10:**
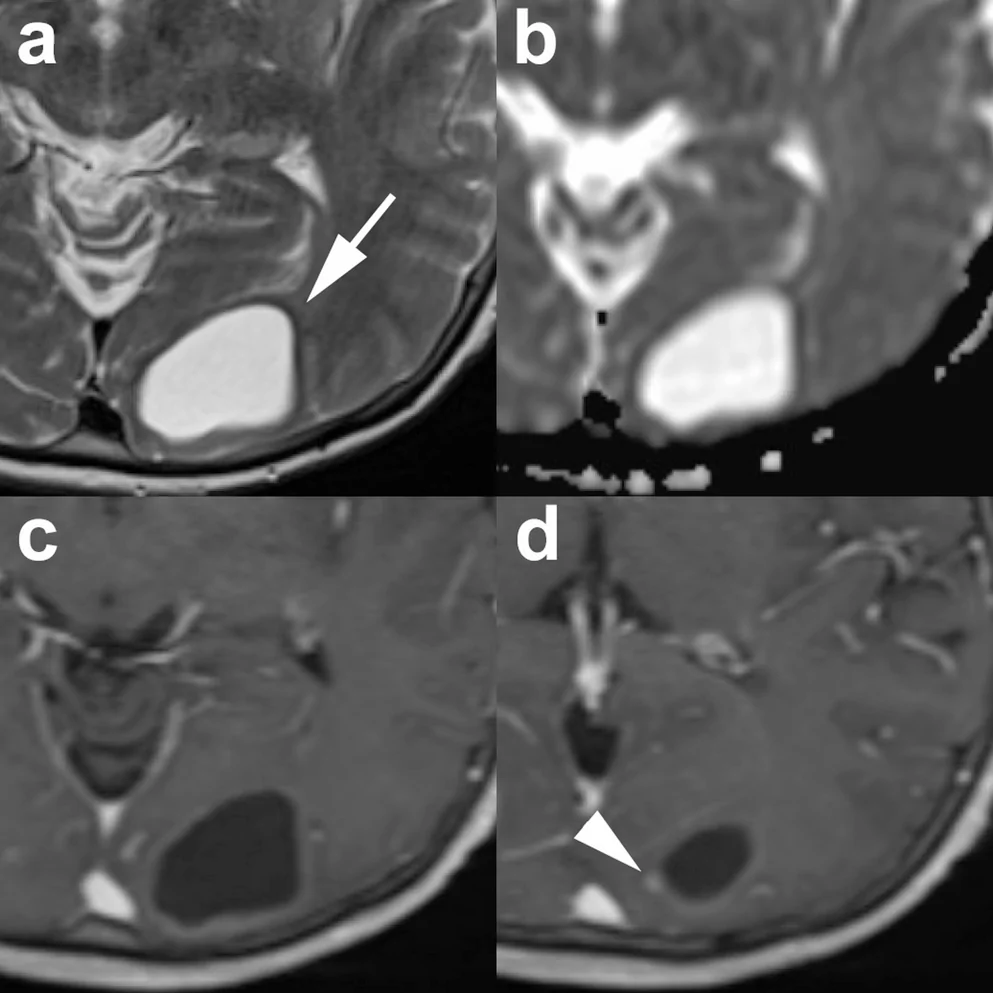
A 69-year-old woman with a history of breast cancer and lung, liver, and bone metastases presented with brain metastases. Axial T2-weighted image (**a**) demonstrates a homogeneous hyperintense cystic mass in the left occipital lobe (arrow). Contrast-enhanced T1-weighted images (**b**, **c**) show slight rim enhancement with a small enhancing mural nodule (arrowhead). The intralesional fluid component demonstrates elevated ADC values (**d**). Multiple additional lesions with solid and cystic components were present in the brain parenchyma (not shown)

These lesions typically appear as ring-enhancing masses with relatively well-defined margins (Fig. 10). On MRI, the cystic component is hypointense on T1-weighted images and hyperintense on T2-weighted images. These cystic components often represent necrotic change. The enhancing rim is seen after gadolinium administration. Extensive vasogenic edema is frequently seen, often out of proportion to the size of the lesion, contributing significantly to mass effect [99]. Although uncommon, calcification has occasionally been described. DWI typically shows no restriction in the cystic core, helping to differentiate from brain abscess. Restricted diffusion may be observed in the enhancing rim depending on tumor cellularity. On PWI, the enhancing rim demonstrates elevated rCBV. Given that brain metastases often occur as multiple lesions, there have been reports of cases mimicking neurocysticercosis [98–100, 105–107]. Notably, brain metastases from ALK-rearranged non-small cell lung cancer treated with crizotinib may present as multiple cystic lesions with minimal enhancement and little surrounding edema [108, 109]. Other differential diagnosis includes high-grade gliomas, abscesses, and tumefactive demyelination. Solitary cystic metastasis may mimic a primary brain tumor, sometimes necessitating histologic confirmation.

Systemic therapy directed at the primary malignancy plays a crucial role in treatment. Management for patients with symptomatic brain metastases includes surgical resection and stereotactic radiosurgery [110]. Whole-brain radiotherapy represents a treatment option in the management of multiple or residual brain metastases.

## Pattern-based approach to cystic brain neoplasms

Here, we propose a structured, pattern-based approach that can guide the diagnostic evaluation of cystic brain tumors (Supplementary Material 1). Cystic brain tumors can be broadly categorized into several morphologic patterns.

First, cystic lesions with an enhancing mural nodule are classically associated with entities such as HB, PA, PXA, ganglioglioma, and DIG/DIA. Second, multicystic or “bubbly” lesions, characterized by multiple small cyst-like spaces, are commonly seen in DNET and central neurocytoma. These lesions often show a cluster of small cystic components with minimal mass effect relative to lesion size. Third, solid masses with cystic components include ependymoma, glioblastoma, and brain metastasis. Ependymoma typically shows well-defined margins with heterogeneous enhancement, whereas glioblastoma often presents as an irregular ring-enhancing mass and metastasis typically appears as a relatively well-circumscribed ring-enhancing lesion.

In addition to these imaging patterns, evaluation of lesion location can further refine the differential diagnosis. Patient age is another critical factor that further constrains the differential diagnosis, as many cystic brain tumors demonstrate characteristic age predilections. Furthermore, advanced imaging techniques provide complementary information regarding tumor characteristics. Accordingly, integration of clinical and imaging features is essential for brain tumor diagnosis.

## Conclusion

Recognizing characteristic imaging features such as the morphology of the cyst, lesion location and enhancement pattern, along with relevant clinical information is critical for narrowing the differential diagnosis. Advanced MR imaging techniques, including diffusion, perfusion, and spectroscopy, further contribute to noninvasive evaluation of lesions. A comprehensive understanding of these imaging characteristics enhances diagnostic confidence and improves patient outcomes.

## Supplementary Information

Below is the link to the electronic supplementary material.Supplementary Material 1
