# Supplementary material for: Imaging diagnosis of cystic intraparenchymal brain neoplasms
Source: Jpn J Radiol. 2026 May 30;44(9):1519–33. doi: 10.1007/s11604-026-02001-w (PMC13525972; doi:10.1007/s11604-026-02001-w)
Supplement: Supplementary file 1 [file 11604_2026_2001_MOESM1_ESM.docx]

**Supplementary Material 1. Diagnostic Flowchart for Brain Neoplasms with Cystic Components**
